# DEGGs: an R package with shiny app for the identification of differentially expressed gene–gene interactions in high-throughput sequencing data

**DOI:** 10.1093/bioinformatics/btad192

**Published:** 2023-04-21

**Authors:** Elisabetta Sciacca, Salvatore Alaimo, Gianmarco Silluzio, Alfredo Ferro, Vito Latora, Costantino Pitzalis, Alfredo Pulvirenti, Myles J Lewis

**Affiliations:** Centre for Experimental Medicine and Rheumatology, William Harvey Research Institute, Barts and The London School of Medicine and Dentistry, Queen Mary University of London, London EC1M 6BQ, United Kingdom; Centre for Translational Bioinformatics, William Harvey Research Institute, Barts and the London School of Medicine and Dentistry, Queen Mary University of London, London EC1M 6BQ, United Kingdom; Department of Clinical and Experimental Medicine, University of Catania, Catania 95123, Italy; Dipartimento di Matematica e Informatica, University of Catania, Catania 95123, Italy; Department of Clinical and Experimental Medicine, University of Catania, Catania 95123, Italy; School of Mathematical Sciences, Queen Mary University, London E1 4NS, United Kingdom; Dipartimento di Fisica ed Astronomia, Università di Catania and INFN, Catania I-95123, Italy; Centre for Experimental Medicine and Rheumatology, William Harvey Research Institute, Barts and The London School of Medicine and Dentistry, Queen Mary University of London, London EC1M 6BQ, United Kingdom; Department of Clinical and Experimental Medicine, University of Catania, Catania 95123, Italy; Centre for Experimental Medicine and Rheumatology, William Harvey Research Institute, Barts and The London School of Medicine and Dentistry, Queen Mary University of London, London EC1M 6BQ, United Kingdom; Centre for Translational Bioinformatics, William Harvey Research Institute, Barts and the London School of Medicine and Dentistry, Queen Mary University of London, London EC1M 6BQ, United Kingdom

## Abstract

**Summary:**

The discovery of differential gene–gene correlations across phenotypical groups can help identify the activation/deactivation of critical biological processes underlying specific conditions. The presented R package, provided with a count and design matrix, extract networks of group-specific interactions that can be interactively explored through a shiny user-friendly interface. For each gene–gene link, differential statistical significance is provided through robust linear regression with an interaction term.

**Availability and implementation:**

DEGGs is implemented in R and available on GitHub at https://github.com/elisabettasciacca/DEGGs. The package is also under submission on Bioconductor.

## 1 Introduction

Unveiling differences between groups of patients is pivotal to healthcare customization. To this aim, modern clinical trials frequently use RNA-sequencing in blood samples or tissue biopsies, enabling comprehensive transcriptomic analyses between different groups.

In this context, identifying differentially expressed genes (DEGs) is one of the most common initial analyses, usually performed via well-known R Bioconductor packages such as DESeq2, edgeR, or limma-voom.

Although the resulting DEGs can suggest possible active processes, it is often difficult to derive complex biological mechanisms from single gene expressions. For this reason, biological pathways are used to visualize and explain how genes/proteins influence each other and lead to specific processes. However, most pathway-based tools return a list of perturbed predefined, standard pathways in which linked genes/proteins are supposed to be co-expressed. Here we introduce a novel R package that statistically validates such a co-expression within the user’s dataset. Starting from 10 537 interactions collected from publicly available pathway repositories (Kanehisa & Goto, 2000; Da Hsu et al., 2011; Xiao et al., 2009; Tong et al. 2019), the DEGGs package extract those edges that are reasonably active within the provided cohort and allows to unveil differential interactions between different phenotypical groups defined by the user. In comparison to a pathway enrichment analysis, this allows to focus on a much lower number of interactions with greater relevance for the studied cohort. A more detailed inspection of the data is provided thanks to the creation of bespoke, group-dependant molecular sub-networks.

Links shown in each sub-network are modelled via robust linear regression. To understand whether the gene–gene relationship is group dependent in the provided cohort of samples, the model formula incorporates an interaction term that enables the discovery of gene–gene correlations that are statistically different among user-defined groups.

Other packages have been designed to discover differential gene-gene correlations in omics data (Fukushima 2013; McKenzie et al. 2016). However, these packages are not designed for next-generation sequencing (NGS) transcriptomic data where large numbers of expressed genes are detectable (20 000–50 000). In this context, the analysis of all possible gene-gene combinations (of the order of 10^9^) would be necessarily confounded by large numbers of false positive gene pairs biologically unrelated. Using a curated interaction network addresses this issue by reducing the number of possible gene-gene interactions to those which are functionally relevant. Additionally, none of the existing packages provide a user-friendly, interactive interface.

## 2 Implementation

**Figure 1.**
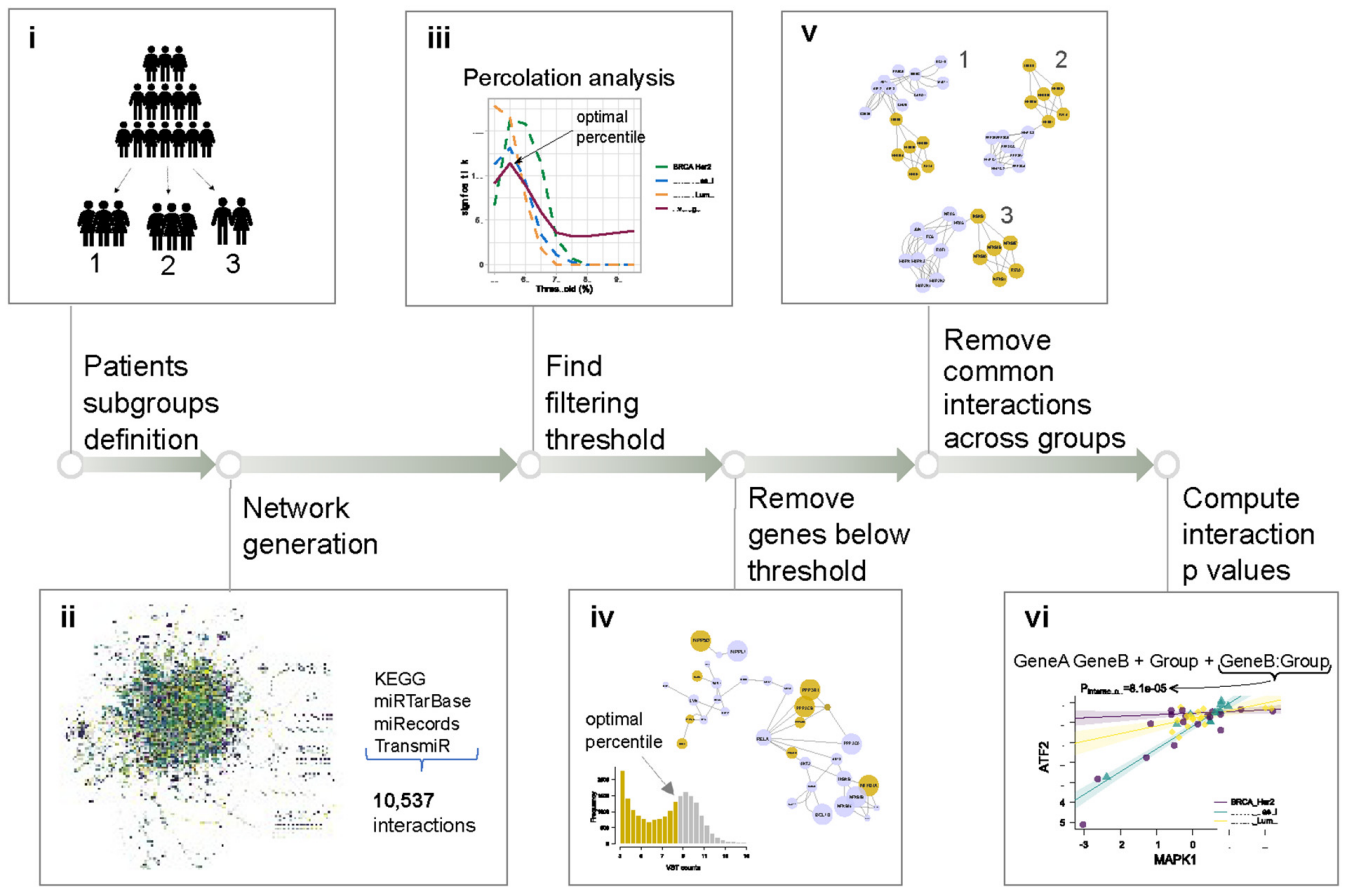
Steps of the analytical pipeline that is internally implemented by the DEGGs package.

The required data for a differential gene–gene expression analysis is a matrix of normalized read counts where genes are organized in rows and samples in columns. Each matrix entry represents the number of sequencing reads mapped to a gene in a sample. Data must be scaled to account for sequencing depth and heteroscedasticity. Along with expression values, a design matrix is also needed to map samples with the subgroups used for differential comparison (Fig. 1, i).

This data are used on an extensive network, called meta-pathway, of 10 537 molecular interactions obtained from KEGG (Kanehisa & Goto, 2000), mirTARbase (Da Hsu et al., 2011), miRecords (Xiao et al., 2009), and transmiR (Tong et al., 2019) (Fig. 1, ii). The meta-pathway has been obtained via the exportgraph function in MITHrIL (Alaimo et al., 2016) and included in the package. First, the molecular interaction network is replicated and annotated for each user-defined group, assigning average gene expression to node weights. Then, two filtering steps are performed to extract group-specific subnetworks. The first step removes nodes with average expression levels below a cut-off set through percolation analysis. Commonly used in statistical physics and mathematics, percolation describes the behaviour of network properties at increasing percentages of removed nodes or links (Sahini & Sahimi, 1994). This implementation optimized the percolation threshold to maximize the number of statistically significant differential interactions (Fig. 1, iii and iv). A second filtering step removes common interactions across groups (Fig. 1, v). Lastly, the statistical significance of the remaining links is evaluated by building a robust linear regression model or a one-way ANOVA when more than two groups must be compared. The model formula explores the relationship between each gene–gene pair incorporating the group variable as an interaction term: where *i *=* *1, …, *n*, is the number of samples and *ε_i_* are random variables.


$${\mathrm{Gene}A}_{i}=\beta_{0}+\beta_{1}{\mathrm{Gene}B}_{i}+\beta_{2}{\mathrm{Group}}_{i}+\beta_{3}{\mathrm{Gene}B}_{i}\;*\;{\mathrm{Group}}_{i}+\varepsilon_{i},$$

*P*-values of the *F*-test on the Gene*Group term assess the statistical differential significance of the link across the examined groups (Fig. 1, vi).

## 3 Case study

To show the package’s functionalities, we use breast cancer expression profiles collected from The Cancer Genome Atlas program (TCGA) (Weinstein et al., 2013). As an example, we compare the HER2-positive and luminal-A breast tumour subtypes.

The raw RNA-seq count data have been normalized via limma-voom and provided in the package:

data("BRCA_metadata")

data("BRCA_normCounts")

To generate the HER2-positive and luminal-A specific networks, the generate_subnetworks function is used. Entrez formatted gene IDs and gene symbols are permitted and controlled via the entrezIDs parameter. When entrezIDs=TRUE, the user can choose whether to show gene symbol IDs in the output. The convert_to_gene_symbols option controls this behaviour.

subnetworks_object <-generate_subnetworks

(normalised_counts = BRCA_normCounts, metadata = BRCA_metadata, subgroup_variable = "SUBTYPE", subgroups = c("BRCA_Her2", BRCA_LumA"), entrezIDs = TRUE, convert_to_gene_symbols = TRUE)

The generate_subnetworks function returns an object of class DEGGs, which contains a list of specific network tables, the total number of statistically significant links, and the input data.

The output of this function can be used to visualize subtype-specific networks and single gene-gene correlations. The View_interactive_subnetwork function can be called to navigate the networks interactively:

View_interactive_subnetwork(subnetworks_object)

**Figure 2.**
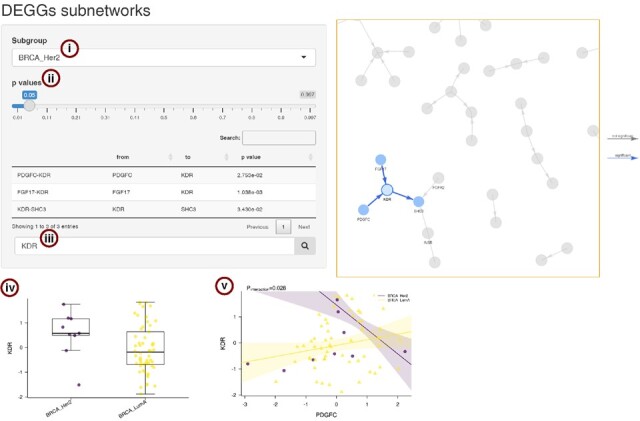
Screenshot of the interactive shiny interface showing (i) the subgroup drop-down menu, (ii) a slider for the visualization of links below a *P* value threshold, (iii) the node search box, (iv) an individual node boxplot, and (v) a link regression plot.

This function allows users to select the generated networks (Fig. 2, i), filter by gene–gene link significance (Fig. 2, ii), and search for specific genes of interest (Fig. 2, iii). When selecting a node, a boxplot comparing gene expression levels between subgroups is shown (Fig. 2, iv), along with a table listing all the gene’s neighbours. When clicking on a link, the differential gene–gene regression model is plotted (Fig. 2, v).

This type of result allows a much greater granularity when compared to pathway enrichment. For example, in the sample data the Ras signaling pathway would have been detected as upregulated for the HER2-positive group without any further information on the links that can be considered as active within it. DEGGs, instead, identifies the links between *KDR* and *FGF17*, *SHC3* and *PDGFC* as active and specific for the HER2-positive group.

Furthermore, the use of gene–gene pairs that show significant differential correlation has been shown to improve predictive models of treatment response in rheumatoid arthritis (Sciacca et al., 2022). The list of significant gene pairs found across networks can be obtained through the extract_sig_deggs function:

extract_sig_deggs(subnetworks_object)

The listed genes can then be used as features of machine learning models.

## 4 Conclusions

Detecting differential gene-gene correlations can shed light on molecular mechanisms that differentiate phenotypical groups. However, due to the large number (20 000–50 000) of expressed genes detectable with NGS techniques, the analysis of all the possible gene-gene correlations (up to 2.5 × 10^9^) is computationally expensive. As a further shortcoming, the false positive rate of gene-gene pairs, which are biologically and functionally unrelated, is high.

The presented package enables the interactive exploration of group-specific networks and finds gene-gene correlations which are statistically different among groups. DEGGs makes use of linear regressions with interaction term to evaluate the differential co-expression between gene expressions, therefore non-linear relations cannot be detected, and this is a limitation of the package.

For linear regressions, the list of identified differential gene–gene pairs can be used as feature selection method in machine learning models.
